# Enhanced Adsorption of Bromoform onto Microplastic Polyethylene Terephthalate Exposed to Ozonation and Chlorination

**DOI:** 10.3390/molecules28010259

**Published:** 2022-12-28

**Authors:** Ximiao Zhu, Chenhui Hao, Mengze Zhang, Bingyan Lan

**Affiliations:** School of Environment, South China Normal University, Guangzhou 510006, China

**Keywords:** polyethylene terephthalate, oxidation process, water/wastewater treatment plant, disinfection by-products, bromoform

## Abstract

This paper selected microplastic polyethylene terephthalate (PET), commonly found in water/wastewater plant effluent, to investigate the changes of PET oxidized under ozonation (designated as ozonized PET), followed by sodium hypochlorite oxidation (designated as ozonized-chlorinated PET) and studied their influence on the adsorption of the disinfection by-product bromoform (TBM). Fragmentation and cracks appeared on the oxidized PET surface. As the oxidation degree increased, the contact angle decreased from 137° to 128.90° and 128.50°, suggesting hydrophilicity was enhanced. FTIR and XPS analyses suggested that carbonyl groups increased on the surface of ozonized PET and ozonized-chlorinated PET, while the formation of intermolecular halogen bonds was possible when PET experienced dual oxidation. These physiochemical changes enhanced the adsorption of TBM. The adsorption capacity of TBM followed the order of ozonized-chlorinated PET (2.64 × 10^−6^ μg/μg) > ozonized PET (2.58 × 10^−6^ μg/μg) > pristine PET (2.43 × 10^−6^ μg/μg). The impact of raw water characteristics on the adsorption of TBM onto PETs, such as the pH, and the coexistence of inorganic ions and macromolecules (humic acid, surfactant, and bovine serum albumin) were studied. A different predominant adsorption mechanism between TBM and pristine PET or oxidized PETs was proposed.

## 1. Introduction

It is reported that approximately 5.70 × 10^4^ to 2.65 × 10^5^ million metric tons of plastic debris are released from global riverine systems annually into the oceans [1] with the increasing consumption of plastic products. The Marine Strategy Framework Directive (2008/56/EC) [2] classifies plastics with a size greater than 25 mm as macroplastics, with a diameter of 5–25 mm—as mesoplastics, with a diameter of less than 5 mm—microplastics, while plastics with a size of less than 1000 nm are nano-plastics. In addition to cosmetics and personal care products, water-based coatings, adhesives, electronic products, etc., will release plastic particles. All of them are sources of primary microplastics (MPs) [3]. Secondary MPs mostly come from the crushing effect of large pieces of plastics under the common effect of weathering, atmospheric oxidation, UV irradiation, and biodegradation [4]. MPs’ apparent morphology and surface chemistry will become more and more diverse under these effects. When MPs act as carriers of pollutants in a water body, the combined pollution behavior of aged MPs and pollutants will be more complicated, and in turn, cause greater ecological risks [5].

The concentration of MP particles in the influent to the wastewater treatment plants (WWTPs) varies between 1.5 × 10^4^ and 9.1 × 10^5^ MPs/m^3^ [6]. Multiple studies have been conducted on the removal of MPs by various treatments. Conley et al. found that the influent received 10,000 MPs/person/day in three investigated WWTPs and approximately 75–99% of MPs were removed through wastewater treatment processes [7]. Despite the high percentages removed, the total number of MPs released to natural bodies of water could be several million per day due to the large volumes of effluent discharged daily.

Natural water bodies are the main accumulation sites of MP pollution. It involves the whole natural water ecosystem, from freshwater to seawater, from water bodies to sediment [8]. MP pollution in rivers varies globally, with an abundance of about 1/m^3^ in mildly polluted rivers while the abundance can reach 1 million/m^3^ in heavily MP-polluted waters [9]. Lakes have also become one of the important areas of MP pollution. The average abundance of MPs in the Great Lakes of North America [10,11,12] is about 2 million/km^2^. In China, the abundance of MPs in Poyang Lake, Taihu Lake, and Dongting Lake is 5 × 10^6^~34 × 10^6^/m^3^, 3.4 × 10^6^/m^3^~25.8 × 10^6^/m^3^, and 900~2800/m^3^, respectively [13,14,15]. Freshwater is the main source of human drinking water. MPs in drinking water treatment plants (DWTPs) are already considered a possible hazard to human health. The removal of MPs by drinking water treatment processes has received growing interest. Pivokonsky et al. investigated three DWTPs in the Czech Republic and found 70% removal efficiency of MPs by coagulation/flocculation and sand filtration, 81% by coagulation/flocculation, sedimentation, sand, and granular activated carbon filtration, and 82% by coagulation/flocculation, flotation, sand filtration, and granular activated carbon filtration [16]. Sarkar et al. investigated a DWTP sourced from the river Ganga, India, achieving 85.3% MPs removal via chlorination, coagulation, pulse clarification, and sand filtration [17]. Wang et al. found that the overall removal efficiency of MPs was 82.1–88.6% in the advanced DWTP using a conventional treatment process combined with ozonation/GAC filtration [18].

It can be seen that both WWTPs and DWTPs are important barriers to reducing the number of MPs from water. However, neither treatment plant can fully cut off MP pollution. The most common polymers in the effluent were PET (polyethylene terephthalate), PP (polypropylene), and PE (polyethylene). MPs smaller than 10 μm were the most plentiful in both raw and treated drinking water samples, accounting for up to 95% [16,19]. In the effluent of WWTPs, >95% of MPs are in the range of 20–300 μm [7]. Particles smaller than 50 μm significantly predominated in most of the tap water samples in China [20].

Various aging processes change MPs, including ultraviolet radiation, physical wear, wind and shear force, chemical oxidation, and biodegradation [21,22]. Kelkar et al. [23] found that PS (polystyrene ~0.05 × 2 × 2 mm) can be chemically altered at chlorine doses characteristic of wastewater disinfection and discovered the formation of Cl-C bonds during chlorination of HDPE (high-density polyethylene) under extreme dosages. Fan et al. simulated the aging behavior of MPs in the natural environment (50 W/m^2^ UVA irradiance, 72 h) [24]. It is found that the specific surface area, surface negative charge, the content of the oxygen-containing functional group, and hydrophilicity of UV-aged MPs increased, therefore increasing the adsorption capacity of antibiotics.

Both ozone and sodium hypochlorite are strong oxidants and play an important role in pre-oxidation and post-disinfection treatment processes. They can degrade toxic and harmful organics, algae, and microorganisms, enhancing the treatment/purification efficiency of WWTPs or DWTPs. On the other hand, they cannot avoid producing oxidation/disinfection byproducts when they interact with natural organic matter and halogen anions, which are ubiquitous in natural waters. Trihalomethanes including chloroform (CHCl_3_), dichlorobromomethane (CHCl_2_Br), chlorodibromomethane (CHClBr_2_), and bromoform (CHBr_3_) are mostly generated during the treatment flow of drinking water [25]. He et al. [26] investigated the two full-scale drinking water facilities in Las Vegas, NV USA, and found the implementation of ozone favored the formation of the di- and tri-brominated species such as bromoform and chlorodibromomethane. THMs are known to have cancer risk and bromoform contributed the highest cancer risk through oral ingestion [27]. Disinfection by-products are generally a drinking water problem and raise a great concern in wastewater reuse processes. When MPs enter the water plant, oxidation by ozone and hypochlorite alters MPs’ physicochemical characteristics. Shao et al. [28] illustrated that oxidation of PE by conventional pro-oxidants (potassium permanganate, sodium hypochlorite, and ozone) changed the wetness, specific surface area, and functional groups of the PE surface. Liu et al. [29] researched the accelerated aging behaviors of polystyrene (PS) and polyethylene (PE) by Fenton and heat-activated K_2_S_2_O_8_ treatments.

To the best of our knowledge, studies examining the oxidation of MPs in the treatment units of WWTPs and DWTPs are still rare, and little is known about the adsorption behavior of oxidized MPs towards oxidation/disinfection by-products generated along with the treatment flow. Oxidation accelerates the aging of MPs. The objective of this study was to simulate the PET particles in the pre-oxidation and post-disinfection process by using ozone and ozone combined with hypochlorite and investigated the oxidation degree of PETs having a size of 13 μm, which are abundant in water treatment effluent. The adsorption behavior of oxidized PETs towards bromoform (TBM) was investigated. Considering the complexity of raw water, we introduced coexisting ions and humic acid to analyze their impacts on adsorption. Moreover, to simulate the daily water use scenario, the influence of oxidized PETs encountering surfactant and bovine serum albumin on TBM adsorption was discussed. The combination of oxidized microplastics and disinfection by-products makes the impact of these two emerging micropollutants on the ecological environment and human health more multifaceted, and the findings of this paper provide new insight into the environmental behavior and ecological risks of microplastics.

## 2. Results and Discussion

### 2.1. Characteristics of Pristine PET and Oxidized PET

**Figure 1:**
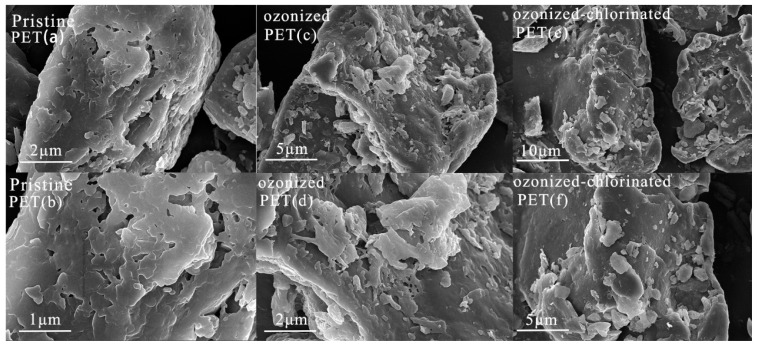
SEM micrograph of pristine PET (**a**,**b**), ozonized PET (**c**,**d**), ozonized-chlorinated PET (**e**,**f**).

**Figure 2:**
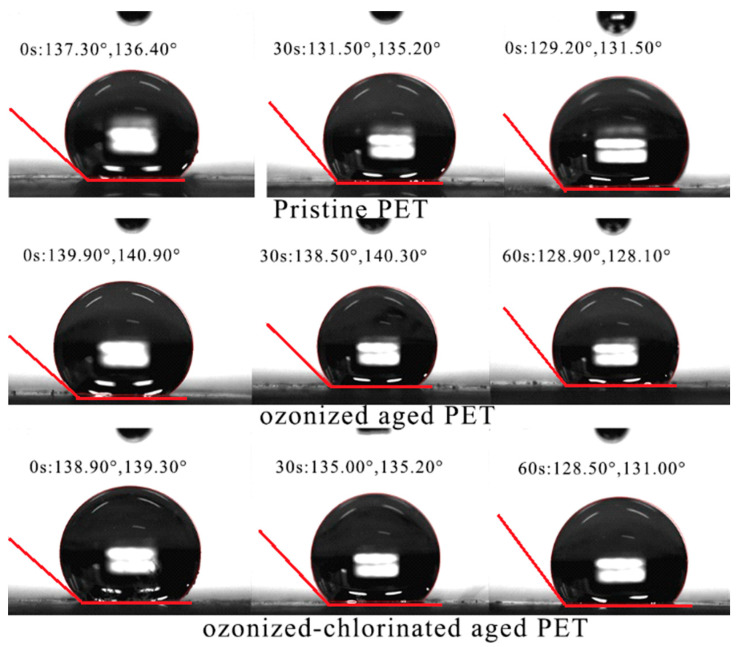
Contact angle (left) of pristine PET, ozonized PET, ozonized−chlorinated PET.

The SEM images (Figure 1) display that pristine and oxidized PET surface structures differ. There were obvious pores and layered structures on the surface of pristine PET, which facilitated the adsorption of organics. Compared with pristine PET, oxidized PETs had more obvious rough surfaces with debris and cracks. Fragmentation suggested the breakage of PET backbones leading to the formation of side chains, oxygenated functional groups, and the release of additives inside PETs. The increase in pore structure directly affected the specific surface area and provided more sites for the adsorption of pollutants. It was reported [30,31] that cracks appearing on the surface of glassy polymer PET after aging have enhanced the surface permeability and decreased the contact angle. From the dynamic contact angle analysis in Figure 2, the left contact angle of different treated PETs after 60 s was in the order of pristine PET(137°) > ozonized PET (128.90°) > ozonized-chlorinated PET (128.50°). The degree of the left contact angle reduced as the time of the oxidation process increased.

**Figure 3:**
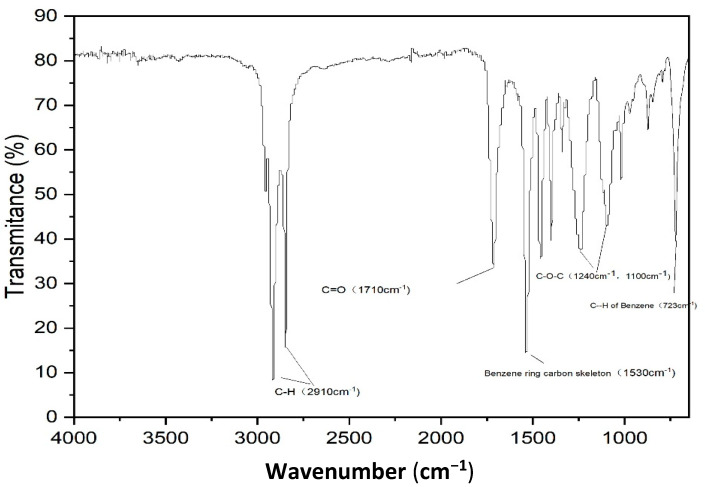
The characteristic FT−IR spectra of the raw PET.

**Figure 4:**
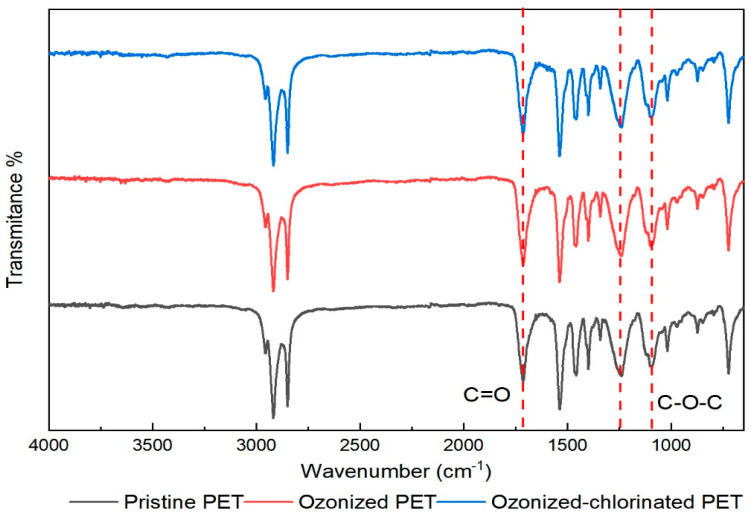
The FT−IR spectra of the PET with different oxidation treatments.

**Figure 5:**
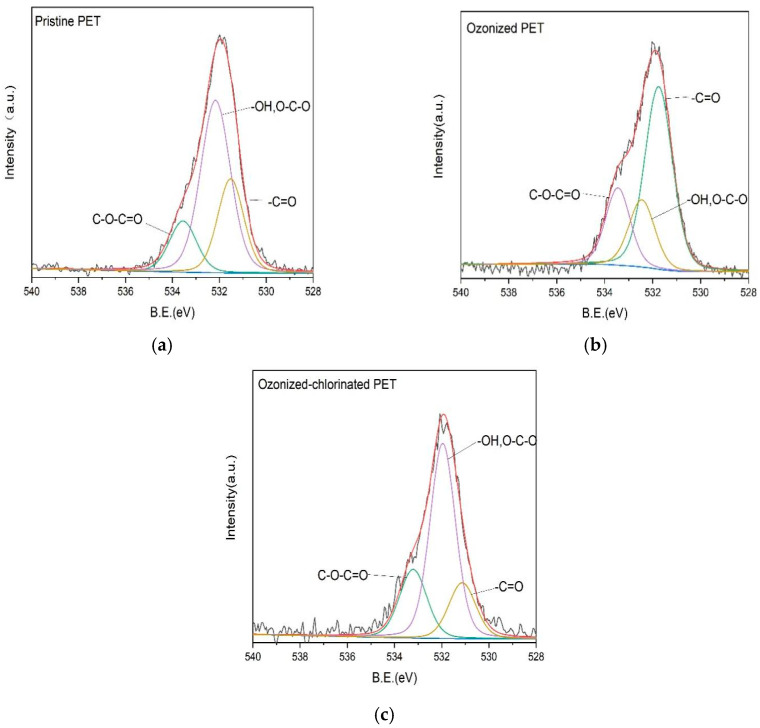
The result of XPS of (**a**) pristine PET, (**b**) ozonized PET and (**c**) ozonized−chlorinated PET before adsorption.

Fourier transform infrared spectra were used to investigate the changes in the chemical properties of pristine and oxidized PETs. The FTIR spectra of pristine PET are shown in Figure 3. Several characteristic peaks of PET were observed. The adsorption peak at 1720 cm^−1^ was caused by the stretching vibration of the carbonyl group (C=O). The 1240 cm^−1^ and 1100 cm^−1^ were the adsorption peak caused by stretching vibration of the ether bond (C-O-C) and carbon-oxygen bond (C-O) of the ester bond, which indicated the existence of esters. The 723 cm^−1^ was the adsorption peak caused by plane bending vibrations of the benzene rings in PET [32]. The oxidation of PET can cause the rupture of the ester bond, forming the carboxylic acid end group and vinyl end group. The FTIR spectra of oxidized PETs are shown in Figure 4. The intensity of oxygenated functional groups such as the carbonyl group and C-O-C and C-O of ester bond was slightly increased. The carbonyl index (CI) is used to quantitatively evaluate MPs’ oxidation degree. It refers to the ratio of the adsorption intensity of the carbonyl group to the adsorption intensity of a reference peak ($\mathrm{Carbonyl}\;\mathrm{index}=\frac{\mathrm{Absorbance}\;\mathrm{carbonyl}\;\mathrm{peak}}{\mathrm{Absorbance}\;\mathrm{reference}\;\mathrm{peak}}$). The reference peak selected in this paper is the hydrocarbon bond at the wavelength of 2850 cm^−1^ according to CI definition [32]. The higher value of CI the higher the oxidation degree of MPs [33,34]. The CI values of pristine PET, ozonized PET, and ozonized-chlorinated PET was 0.818, 0.897, and 0.877, respectively. Furthermore, we investigated the XPS of PET with different oxidation processes as shown in Table 1 and Figure 5. The binding energy of the first peak on the spectrum is 531.1–531.7 eV, which is derived from the carbonyl group of PET. The binding energy of the second peak on the spectrum is 531.9–532.4 eV derived from the hydroxyl groups and O-C-O groups of PET. The binding energy of the third peak on the spectrum is 533.2–533.8 eV derived from the single-bonded oxygen in ester groups. Compared with pristine PET, the content of the carbonyl group and ester groups of ozonized PET increased by 31.04% and 6.27%. The content of hydroxyl and O-C-O groups of ozonized PET decreased by 37.31%. While the content of hydroxyl and O-C-O groups and ester groups of ozonized-chlorinated PET increased by 2.17% and 7.03%. The content of the carbonyl group of ozonized-chlorinated PET decreased by 9.19%. To sum up, carbonyl groups on the ozonized PET surface were more than those on the ozonized-chlorinated PET. After exposure to ozone, the PET chain broke, and cracks formed. When re-oxidized by sodium hypochlorite, the benzene ring of PET may combine chlorine atoms to form C-Cl/πbonds instead of oxygenated functional groups. However, as inspected by FTIR results, there was no new peak after oxidation by ozone and hypochlorite indicating there was no formation of new chemical bonds. Therefore, we speculated the formation of intermolecular halogen bonds which is analogous to hydrogen bonds.

### 2.2. Adsorption TBM on PET

**Figure 6:**
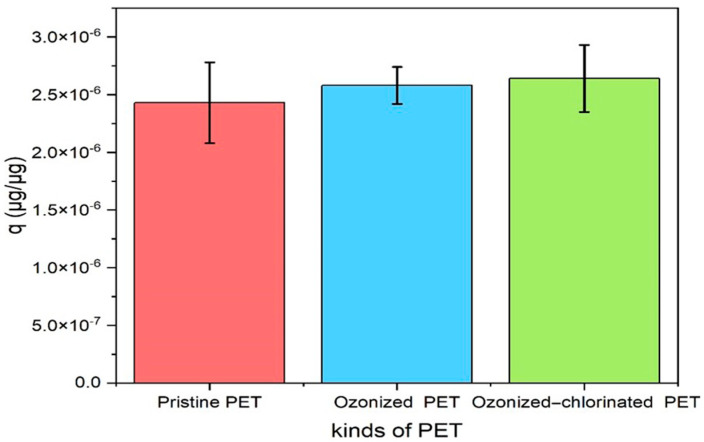
The adsorption capacity of the three PETs on TBM after 24 h.

**Figure 7:**
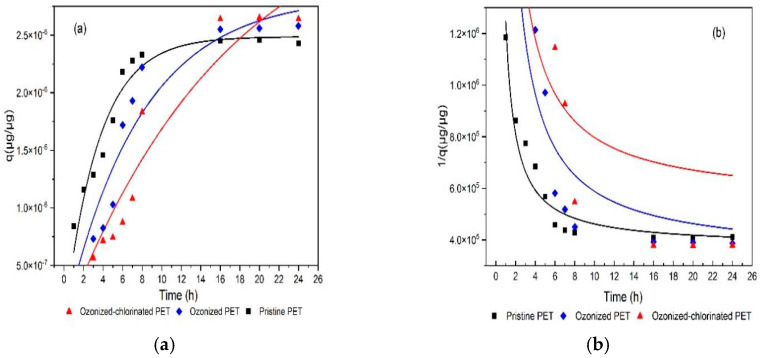
Kinetics model of TBM adsorption by pristine PET, ozonized PET, and ozonized−chlorinated PET. (**a**) Pseudo−first−order model, (**b**) pseudo−second−order model.

The adsorption capacity of TBM on three types of PETs is compared in Figure 6. After 24 h, the adsorption capacity of TBM on PET followed the order of ozonized-chlorinated PET (2.64 × 10^−6^ μg/μg)> ozonized PET (2.58 × 10^−6^ μg/μg)> pristine PET (2.43 × 10^−6^ μg/μg). Figure 7a,b is kinetic model fitting. The adsorption process of TBM onto pristine PET and oxidized PET can be divided into three stages. The first stage was the fast adsorption stage before 10 h. In the second stage, the adsorption rate slowed down from 10 h to 16 h. In the last stage, the adsorption equilibrium was achieved within 24 h. The kinetic parameters simulated from the pseudo-first-order model and pseudo-second-order model are listed in Table 2. The pseudo-first-order model can better describe the adsorption behaviors (R^2^ = 0.9234–0.9381, *p* < 0.01). Pristine PET was the fastest to reach adsorption equilibrium, but the adsorption capacity of ozonized-chlorinated PET was the highest. Oxidized PET had a higher adsorption capacity compared with pristine PET contributing to its fragmentation and exposed surface functional groups [35].

**Figure 8:**
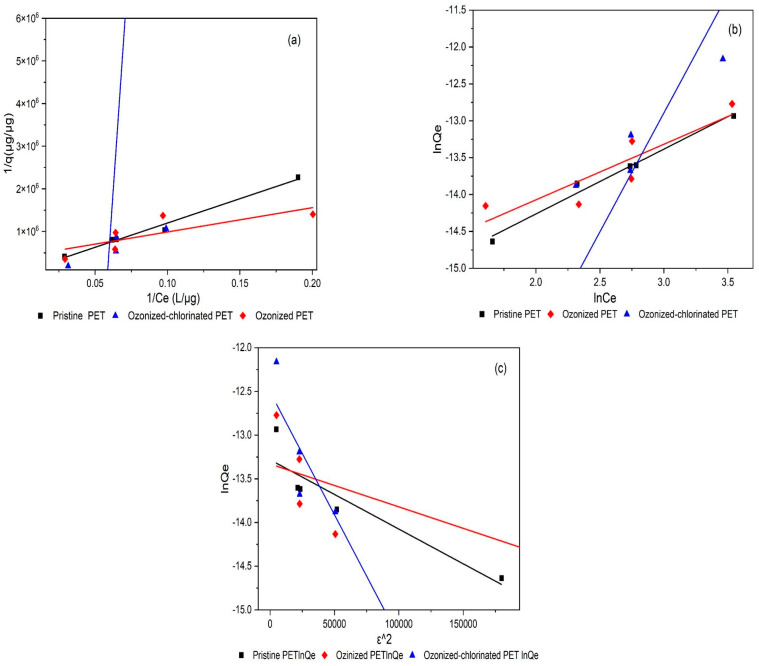
Sorption isotherms of TBM on pristine PET, ozonized PET, and ozonized−chlorinated PET: (**a**) Langmuir model, (**b**) Freundlich model, (**c**) D−R model.

The sorption Isotherms of TBM on PET with the various oxidation modes are shown in Figure 8. The sorption isotherms were measured at pH 7 and 20 °C. The Freundlich, Langmuir, and D-R isotherms models were used to describe the experimental isotherms and isotherm constants. The fitting parameters are summarized in Table 3. As the results shown in Figure 8 and Table 3, with the increased concentration of TBM, the amount of TBM adsorbed by pristine and oxidized PET showed a non-linear rising trend. It is possible that the adsorption was affected by hydrophobic partitioning and other adsorption mechanisms such as van der Waals force and electrostatic interaction.

The pristine PET had better fitting goodness for the Langmuir model (R^2^ = 0.9835, *p* < 0.01), showing that the pristine PET adsorption of TBM was mainly single-layer adsorption due to its hydrophobicity. Kong et al. [36] found that the adsorption mechanism of MPs adsorbing hydrophobic organic pollutants such as antibiotics is mainly the hydrophobic effect. However, ozonized PET was more suitable for the Freundlich model (R^2^ = 0.7228, *p* > 0.01). The Freundlich model is used to describe the adsorption on an uneven surface and multi-layer adsorption. After ozone oxidation, due to the increasing amount of oxygenated functional groups and cracks, the distribution of adsorption sites and active surface functional groups on the surface was uneven. While ozonized-chlorinated PET had the best fitting for the D-R model (R^2^ = 0.9660, *p* < 0.01). It proves that the adsorption of TBM by ozonized-chlorinated PET was heterogeneous. The surface of ozonized-chlorinated PET had more pore structures than the other two types of PET. This can be attributed to the pore-filling adsorption mechanism leading to the uneven distribution of energy at the adsorption sites.

### 2.3. Effects of Water Quality on TBM Adsorption

#### 2.3.1. Effects of pH

**Figure 9:**
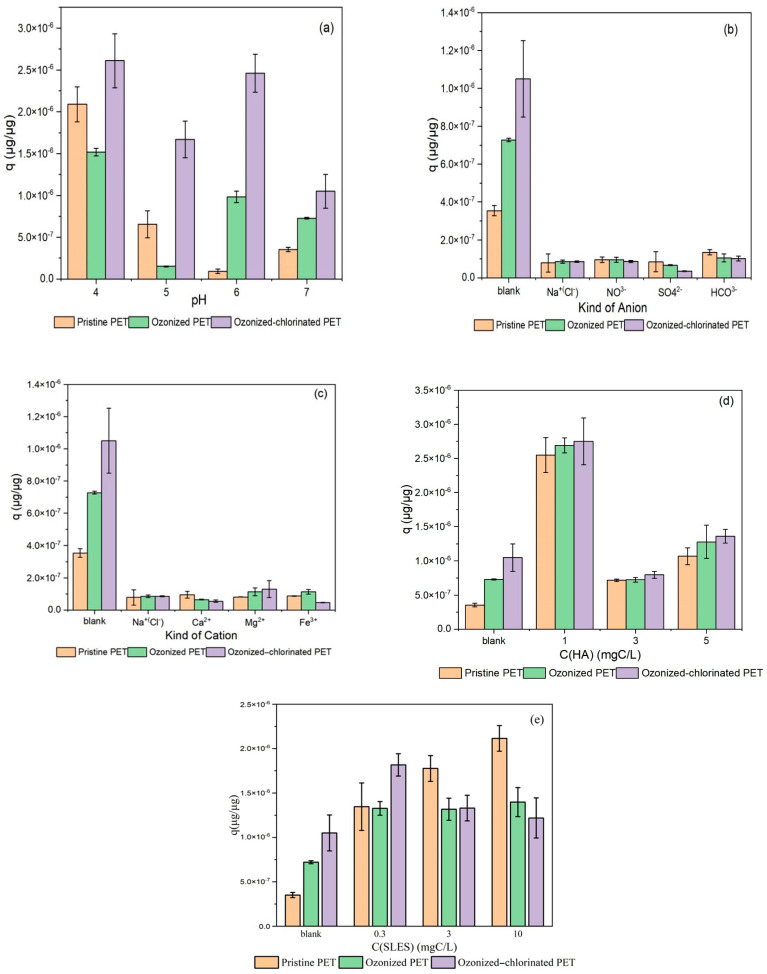
The influence of (**a**) pH, (**b**) kind of anion, (**c**) kind of cation, (**d**) humic acid concentration, (**e**) anion surfactant concentration on TBM sorption to PET with the different oxidation processes. The initial concentration of TBM was 10 μg/L. All the adsorption experiments were conducted at pH = 7 and 20 °C.

The pristine and oxidized PET had various performances when the pH changed from 4 to 7. As an important property in the aquatic environment, pH can change the surface charge of MPs to affect the adsorption capacity of MPs [37]. It has been proved that the outer surface of PET has a nucleophilic nature [38]. Moreover, TBM hydrolyzed in water, which is the process of nucleophilic substitution [39] leading to TBM with a positive charge. Hence, the adsorption mechanism between PET and TBM might include electrostatic interaction. The influence of pH on the adsorption of TBM on pristine PET, ozonized PET, and ozonized-chlorinated PET is shown in Figure 9a. We found that the adsorption capacity came to the highest no matter which type of PET (q_pristine PET_ =2.09 × 10^−6^ μg/μg, q_ozonized PET_ =1.52 × 10^−6^ μg/μg, q_ozonized-chlorinated PET_ = 2.67 × 10^−6^ μg/μg) when pH 4 indicating that low pH was more conducive to the adsorption of TBM on PET through hydrophobic effect [29]. Pristine PET, ozonized PET, and ozonized-chlorinated PET had the lowest adsorption capacity (q_pristine PET_ = 9.34 × 10^−8^ μg/μg, q_ozonized PET_ =7.28 × 10^−7^ μg/μg, q_ozonized-chlorinated PET_ = 1.05 × 10^−6^ μg/μg) at pH = 6, 5, and 7, respectively, indicating that oxidation changed the surface charge density which is related to the zero point of charge of PET. When the pH of the aqueous solution was lower than the zero point of charge of PET, the adsorption capacity of PET reached the lowest because the charge of the PET surface turned out to be positive, consequently, the repulsion between PET and TBM reached the highest [40].

#### 2.3.2. Effects of Inorganic Ions

There are various inorganic ions in the natural aquatic environment. They can affect the adsorption capacity of PET by changing the charge on its surface. The effect of four cations and four anions on the adsorption of TBM on pristine and oxidized PETs is shown in Figure 9a,b. For pristine PET, the adsorption of TBM with NO_3_^−^, SO_4_^2−^, HCO_3_^−^, Na^+^/Cl^−^, Ca^2+^, Mg^2+^, and Fe^3+^ decreased by 73.1%, 76.2%, 61.9%, 77.8%, 73%, 76.9%, and 75.5%, respectively. For ozonized PET, the adsorption of TBM with NO_3_^−^, SO_4_^2−^, HCO_3_^−^, Na^+^/Cl^−^, Ca^2+^, Mg^2+^, and Fe^3+^ decreased by 87.0%, 90.8%, 85.6%, 88.2%, 91%, 84.3%, and 84.5%, respectively. For ozonized-chlorinated PET, the adsorption of TBM with NO_3_^−^, SO_4_^2−^, HCO_3_^−^, Na^+^/Cl^−^, Ca^2+^, Mg^2+^, and Fe^3+^ decreased by 91.7%, 96.6%, 90.3%, 91.8%, 94.7%, 87.6%, and 95.5%, respectively. The presence of anions compressed the electronic double layer on the PET surface so that it inhibited the electrostatic attraction between PET and TBM, while cations competed with TBM for adsorption sites. It shows that inorganic ions inhibited the adsorption more significantly for oxidized PET than pristine PET as oxidation enhanced the hydrophilicity of PET. Carbonate has a small inhibitory effect since bicarbonate can balance the charge in the solution to form a buffer system [38].

#### 2.3.3. Effects of Humic Acid

Humic acid is a natural organic matter, which is a complex and heterogeneous mixture existing in the aquatic environment. It can not only be adsorbed by microplastics but also affect the adsorption of other organic pollutants by microplastics [41,42]. The effects of HA on the adsorption of TBM on pristine PET and oxidized PET is shown in Figure 8d. When the concentration of HA was 1 mg/L, the adsorption of TBM on pristine PET, ozonized PET and ozonized-chlorinated PET rose by 620%, 269.5%, and 161.9%, respectively. When the concentration of HA was 3 mg/L, except that the adsorption of TBM on pristine PET rose 102.3%, the adsorption of TBM on ozonized PET and ozonized-chlorinated PET decreased by 0.552% and 24.1%, respectively.

The molecular size of humic acid is greater than that of TBM, so it is preferentially adsorbed on the surface of microplastics, such as by hydrophobic actions and hydrogen bonding interactions. After coating on the surface of PET, HA which is rich in negatively charged functional groups (e.g., carboxyl and hydroxy groups), can increase the negative surface charges on PET [43]. Electrostatic interactions between TBM and PET were strengthened. Therefore, an obvious boost of adsorption occurred on the pristine PET. The enhanced hydrophilicity of oxidized PET not only reduced the hydrophobic effect with humic acid but produced electrostatic repulsion. Furthermore, fragmentation led to a smaller size of oxidized PET. Humic acid created steric resistance between oxidized PET and TBM, hence hindering the adsorption. When the addition of humic acid increased, this inhabitation effect was more pronounced.

#### 2.3.4. Effects of Anion Surfactant

SLES used in this study did not reach its critical micelle concentration (CMC) within the range of concentrations tested so that micelle did not form and SLES was free and evenly distributed in solution as molecules. As shown in Figure 9e, SLES as an anionic surfactant promoted the adsorption of TBM on pristine and oxidized PET. For pristine PET, with the concentration of SLES at 0.3 mg/L, 3 mg/L, and 10 mg/L, the adsorption of TBM rose by 281.3%, 402.8%, and 498.9%. For ozonized PET, the adsorption of TBM rose by 1726%, 1713%, and 1465.9%. As for ozonized-chlorinated PET, the adsorption of TBM rose by 73.3%, 26.7%, and 16.2%. It solubilized a certain amount of TMB hence promoting the TBM adsorption on all tested PET. SLES adsorbed on the surface of PET as monolayers through hydrophobic interaction, and the hydrophilic group was oriented towards the solution. Due to the negative charge of the hydrophilic end of SLES, TBM could be adsorbed by electrostatic attraction [44]. Therefore, the adsorption capacity of pristine PET increased. For ozonized PET, the above effects are greatly enhanced due to surface fragmentation and the increase of specific surface area after ozone oxidation. For ozonized-chlorinated PET, this promotion effect was not obvious. One reason is that the adsorption mechanism of TBM by ozonized-chlorinated PET was mainly pore filling. On the other hand, ozonized-chlorinated PET had the strongest hydrophilicity. The presence of halogen bonds changed the charge distribution and strengthen its anion effect, and electrostatic repulsion occurred between SLES and part of the ozonized-chlorinated PET surface.

#### 2.3.5. Effects of BSA

**Figure 10:**
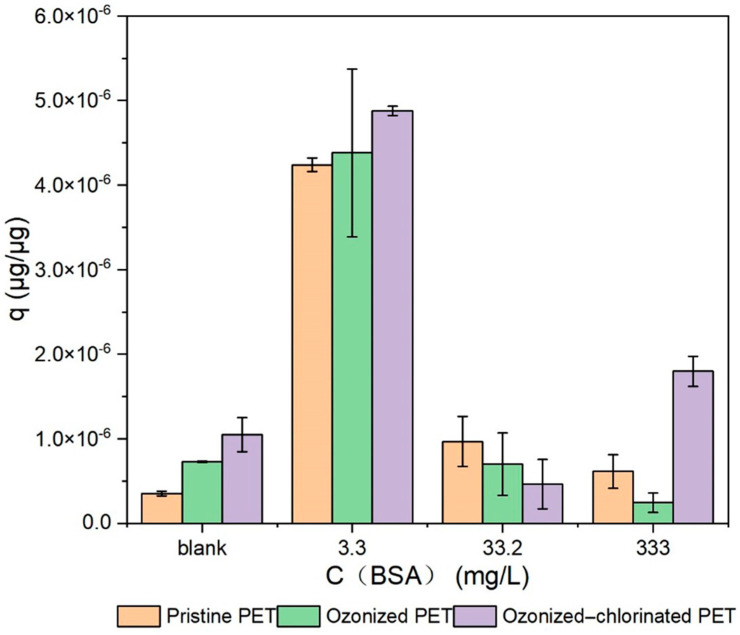
The influence of BSA on TBM sorption to PET with the different oxidation process.

The effect of the presence of BSA on the adsorption of PET toward TBM was shown in Figure 10. At different concentrations of BSA—at 3.3 mg/L, 33.2 mg/L, and 333 mg/L—the adsorption of TBM on pristine PET was promoted by 1097.7%, 173.40%, and 74%, respectively. For ozonized PET, when the concentration of PET was at 3.3 mg/L, the adsorption of TBM was promoted by 501.6%, but for concentrations at 33.2 mg/L and 333mg/L, the adsorption of TBM was reduced by 3.71% and 65.9%, respectively. For ozonized-chlorinated PET, when the concentration of BSA was at 3.3 mg/L and 333mg/L, the adsorption of TBM was promoted by 74% and 71.4%, respectively, but for the BSA concentration at 33.2 mg/L, the adsorption of TBM was reduced 65.9%. It can be seen that the addition of low-concentration BSA (3 mg/L) greatly promoted the adsorption of TBM by three PETs. When BSA was combined with pristine PET, the hydrophilicity of pristine PET was strengthened. The polar ends of BSA, such as amino acid residues, exposed and interacted with TBM by electrostatic adsorption [45]. However, when the hydrophilicity was enhanced after PET oxidation, this promoting effect attenuated. When the concentration of BSA increases, the globular structure of BSA can cause steric repulsion between particles. In addition, BSA also competes with TBM for effective adsorption sites. Therefore, with the increasing amount of BSA, the promoting effect on the adsorption of TBM changes from not obvious to inhibition. It should be noted that the adsorption of TBA on ozonized-chlorinated PET increased by 71.4% when the concentration of BSA was at 333 mg/L. Halogen bonds brought greater electronegativity to the surface of ozonized-chlorinated PET and increased the adsorption of TBM. On the other hand, it is also reported that the presence of Cl ions changes the configuration and the local charge of BSA [46,47].

### 2.4. Adsorption Mechanism

**Figure 11:**
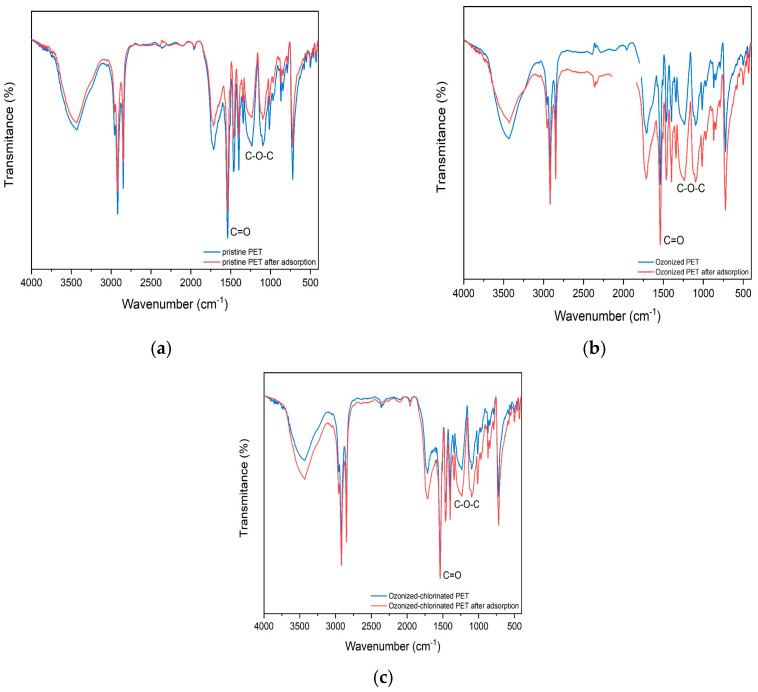
The FT−IR spectra of the PET with different treatments before and after absorption (**a**) pristine PET, (**b**) ozonized aged PET, (**c**) ozonized−chlorinated aged PET.

**Figure 12:**
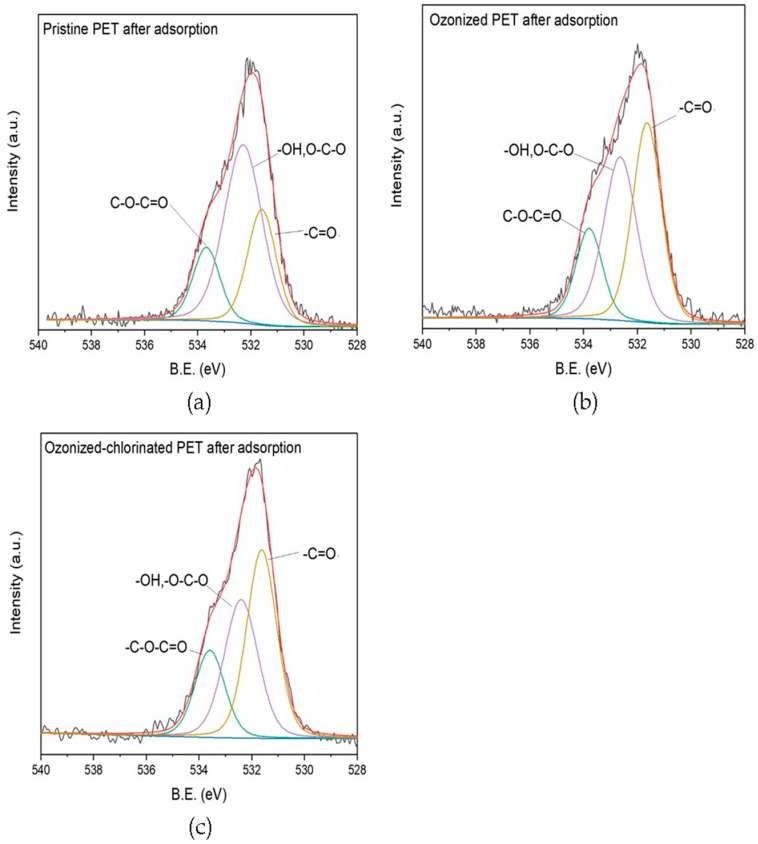
The result of XPS of (**a**) pristine PET, (**b**) ozonized PET and (**c**) ozonized-chlorinated PET after adsorption.

The comparison of FTIR spectra between various treated PET before and after adsorption is shown in Figure 11. It illustrates that no new functional group peak appeared after adsorption, which indicates that no chemical bonding formation occurred during the adsorption process. It can be concluded that the adsorption between various treated PETs and TBM is not predominant chemical adsorption. After the adsorption of TBM onto pristine PET, the stretching vibration of the hydroxyl group located in 3435 cm^−1^ decreased indicating the formation of hydrogen bonds which were converted from hydroxyl groups. Therefore, hydrogen bonding between TBM and pristine PET is involved in the adsorption process. FTIR spectra of oxidized PETs after adsorption shows several discernible changes. After TBM adsorption, the strength of the main functional groups is enhanced, e.g., the peak of the C=O groups, C-O-C groups, and C-O bonds of ester groups. The content of surface hydroxyl groups increased owing to the increased surface wetness. Infrared absorption peaks of oxygenated function groups on the oxidized PET shifted towards lower frequencies due to the increased polarity of carbonyl groups. For a better understanding of the role of oxygen-containing groups in the adsorption process, we investigated the XPS O_1S_ of three PETs after adsorption as presented in Figure 12. The content of oxygenated groups of the pristine PET before and after the adsorption of TBM had no significant difference, demonstrating the main adsorption mechanism between TBM and pristine PET was hydrophobic interactions and hydrogen bonding between molecules. Oxidation and subsequent release of plastic additives from cracks resulted in an increased number of oxygenated groups [48], which caused the reinforcement of electrostatic interactions on the oxidized PET surface. Revealed by XPS spectra, the peak at 531 eV corresponding to the carbonyl groups of the ozonized PET was decreased, which is ascribed to the nucleophilic reactions between hydrolyzed TBM and ozonized PET surface. It is noted that the amount of carbonyl group of ozonized-chlorinated PET increased indicating its adsorption mechanism was dissimilar to ozonized PET. The main adsorption active sites were not the oxygenated functional groups but the intermolecular halogen bonds. Pore filling, electrostatic interactions caused by halogen bonds, and hydrophobic partitioning were the main adsorption mechanisms for ozonized-chlorinated PET.

## 3. Materials and Methods

### 3.1. Materials and Regents

Polyethylene terephthalate (PET), whose size is about 1000 mesh (from Huachuang Plastic Chemical Co., Ltd., Dongguan, China), was ultrasonically cleaned in the 50% ethanol solution and deionized water for 10 min and dried at 30 ℃ for 12 h. Tribromomethane (standard for GC) was obtained from Aladdin Biochemical Technology Co., Ltd. (Shanghai, China). *Tert-Butyl* methyl ether (MTBE, spectrophotometric grade, 99.9%, GC), lauryl ether sulfate sodium (SLES, 70%), bovine serum albumin (BSA), and humic acid (FA ≥ 90%) were bought from Macklin Biochemical Co., Ltd. (Shanghai, China). Sodium sulfate anhydrous (Na_2_SO_4_, AR), sodium hydroxide (NaOH, AR), and iron chloride hexahydrate (FeCl_3_·6H_2_O, AR) were acquired from Zhiyuan Chemical Reagent Co., Ltd., Tianjin, China. Sodium chloride (NaCl, AR) and magnesium chloride hexahydrate (MgCl_2_·6H_2_O, AR) were obtained from Sinopharm Chemical Reagent Co., Ltd. (Shanghai, China). Sodium hypochlorite solution (NaClO, AR), calcium chloride anhydrous (CaCl_2_, AR), and sodium bicarbonate (NaHCO_3_, AR) were bought from Damao Chemical Reagent Factory, Tianjin, China. Sodium nitrate was obtained from Fuchen Chemical Reagent Factory, Tianjin, China. All the glassware was immersed in 5% HNO_3_ solution for 24 h, then rinsed three times with deionized water.

### 3.2. Oxidation of PET

The concentration of PET during the oxidation was about 1 g/L. In order to simulate the oxidation process in a drinking water treatment plant, after ultrasound bathed for 30 min for dispersion, ozone (0.5 g O_3_/h, 2.5 L/min) produced by an ozone generator (CF-G-5G/H, KSSTE, made in China) was passed into the gas-washing bottle. After 15 min, PET was filtered by a vacuum filter, washed twice with deionized water and dried at 30 °C for 12 h.

Ozone/NaClO oxidation process was based on the ozonation process. After 15-min reaction with ozone, the solution with PET suspended was poured into a 500 mL beaker. The oxidation process continued in the solution with NaClO (8 mg Cl^−^/L) on a magnetic stirrer at a high speed for 30 min. Finally, PET was filtered by a vacuum filter, washed twice with deionized water, and dried at 30 °C for 12 h.

### 3.3. Adsorption of TBM on PET

In the kinetic sorption experiment, 1 g/L of different treated PET (pristine PET, ozonized PET, and ozonized/chlorinated PET) mixed with 10 μg/L TBM solution was added to a 40 mL brown glass bottle with a PTFE cap eliminating headspace during adsorption. Samples were collected at different times (1, 2 3, 4, 5, 6, 7, 8, 16, 20, and 24 h) and filtered by a 0.45 μm nylon membrane filter.

In the sorption isotherm experiment, 1g/L of different treated PET (pristine PET, ozonized PET, and ozonized/chlorinated PET), respectively, were mixed with different-concentration TBM solution (5, 10, 15, 20, 25 μg/L). One M NaOH and HCl was used to adjust the pH of the solution (pH = 7). Samples were collected at 24 h and filtered by a 0.45 μm nylon membrane filter.

All of the samples were shaken at 120 rpm at 20 °C. For each experiment, triplicate aliquots were analyzed to obtain an average concentration.

### 3.4. Effect of Water Quality on Adsorption

To study the effects of pH on the adsorption of different treated PET, the initial pH was adjusted to a range from 4 to 7. Inorganic ions and organic macromolecules in the raw water might influence the adsorption of TBM on pristine PET and oxidized PET. Humic acid (FA ≥ 90%) was used as background natural organic matter (NOM) at three different concentrations (1, 3 mg/L) to investigate the effect of NOMs on adsorption. Seven kinds of 0.1 mmol/L electrolytes (NaCl, CaCl_2_, MgCl_2_, FeCl_3_, Na_2_SO_4_, NaNO_3_, NaHCO_3_) were used to investigate the influence of inorganic ions on adsorption. Three different concentrations of SLES solution (0.3, 3, 10 mg/L) were added to the adsorption system. In order to find out the interaction among protein, TBM, and different treated PET, BSA solutions (3.3, 33, 333 mg/L) were introduced into the adsorption system. The initial TBM concentration was fixed at 10 μg/L and PETs were 1 g/L. The initial pH was set to 7. Samples were shaken at 120 rpm at 20 °C. They were collected at 24 h and filtered by a 0.45 μm nylon membrane filter. For each experiment, triplicate aliquots were analyzed to obtain an average concentration.

### 3.5. Characterization of PET

A variety of characterization methods was adopted to characterize pristine PET and oxidized PET. To discover the relationship between the absorption capacity and microscopic morphology of different treated PETs, a scanning electron microscope (SEM, Zeiss Sigma 300, Carl Zeiss) was used to investigate their surface morphologies. Fourier transform infrared spectroscopy (FTIR, TENSOP 37, Bruker) and X-ray photoelectron spectroscopy (XPS, K-Alpha, Thermo Fisher Scientific) were used to measure the functional groups of PETs before and after adsorption of TBM. The contact angle of pristine PET and oxidized PET was tested by contact angle measurement (VCA Optima XE, American stress technologies).

### 3.6. Data Analysis

The concentration of TBM before and after the adsorption procedure were both measured by using gas chromatography (2014C, Suzhou Shimadzu). After stabilization, 1.8 mL of the MTBE supernatant layer was transferred to a 2.0 mL auto-sampler vial and sealed with a Teflon-lined cap for the detection of volatile DBPs by gas chromatography with an electron capture detector (7890A, Agilent). An HP-5MS capillary column (30 m × 0.25 mm with 0.25 µm film thickness, Agilent J&W GC Columns) was used to separate volatile DBPs by following the temperature programming: a hold at 40 °C for 10 min, a ramp to 65 °C at 2.5 °C/min, a ramp to 85 °C at 10 °C/min, a ramp to 205 °C at 20 °C/min, a hold at 205 °C for 7 min. The detector temperature was set at 300 °C and the makeup gas was nitrogen with a flow rate of 1.47 mL/min.

The amount of TBM ions adsorbed per unit mass of pristine and oxidized PET microplastics could be calculated by:

$$Q_{t}=\frac{\left(C_{0}-C_{t}\right)V}{m}$$

$$Q_{e}=\frac{\left(C_{0}-C_{e}\right)V}{m}$$

where m is the mass of pristine and oxidized PET microplastic used in the adsorption procedure (μg), V is the volume of the solution (L), C_0_ and C_e_ are the concentration of TBM before and after the adsorption procedure (μg/L), respectively.

The pseudo-first-order model (PFOM) and pseudo-second-order model (PSOM) were used to describe the sorption kinetics. The PFOM and PSOM can be expressed as:

$$\ln\left(q_{e}-q_{t}\right)=\ln q_{e}-K_{1}t\;\mathrm{or}\;q_{t}=q_{e}\;\times \;(1-e^{{-K}_{1}}t)$$

$$\frac{t}{q_{t}}=\frac{1}{K_{2}q_{e}^{2}}+\frac{t}{q_{e}}$$

where q_t_ (μg/μg) and q_e_ (μg/μg) are the concentrations adsorbed on microplastics at t time and equilibrium and K_1_ (/h) and K_2_ [μg/(μg·h)] are the rate constants of PFOM and PSOM, respectively.

Langmuir, Freundlich, and Dubinin-Radushkevich models were applied to fit the sorption isotherm. Langmuir model can be represented by the following equation:

$$\frac{C_{e}}{q_{e}}=\frac{1}{q_{m}b}+\frac{C_{e}}{q_{m}}$$

where C_e_ is the concentration of TBM when the absorption comes to balance, qm is the maximum TBM uptake of the adsorbents, and b is the Langmuir constant associated with the adsorption rate. The Freundlich equation is described as follows:

$$q={kC_{e}}^{\frac{1}{n}}$$

where K [(μg/μg)/(μg/L)N] is the Freundlich sorption coefficient and N is the exponential coefficient. The Dubinin-Radushkevich equation is described as follows:

$${\mathrm{lnQ}}_{e}={\mathrm{lnQ}}_{\max}-k\cdot \varepsilon^{2}$$

$$\varepsilon =R\cdot T\cdot \ln\left(1+\frac{1}{C_{e}}\right)$$

$$E={\left(2\cdot K\right)}^{-0.5}$$

where Q_max_ [μg/μg] is the maximum absorption capacity, k is Dubinin-Radushkevich constant associated with the adsorption rate and ε is Polanyi potential energy of the absorption process.

### 3.7. Statistical Analysis

Statistical analysis was performed using IBM SPSS statistics version 16.0. One-way analysis of variance (ANOVA) was used to measure the significance of differences (*p* < 0.05).

## 4. Conclusions

In summary, this paper revealed the oxidation of PET under a similar dose and time used in the water/wastewater plant and the induced changes of adsorption behavior for TBM, which was generated along with the oxidation process. After oxidation by ozone or ozone combined with hypochlorite, the hydrophilicity, and micromorphology of the PET surface were altered, and the oxygen-containing functional groups were increased. The adsorption capacity of TBM onto oxidized PET was enhanced, following the order of ozonized-chlorinated PET > ozonized PET > pristine PET. From the perspective of water quality complexity, the coexistence of ions and a high dose of humic acid reduced the adsorption of TBM. From different water usage scenarios, SLES enhanced the electrostatic attraction between TBM and different treated PET and BSA at low doses boosted the hydrophilicity of PET surface thus increasing the adsorption of TBM onto pristine PET and oxidized PET however BSA at high dose generated significant spatial steric hindrance. It is noted intermolecular halogen bonds changed the configuration of BSA and the electronegativity of ozonized-chlorinated PET resulted in increases in TBM adsorption even at a high dose of BSA. FTIR and XPS analyses demonstrated that for non-oxidized PET, the adsorption of TBM was mainly due to the hydrophobic partition while for oxidized PET the surface hydrophilicity, cracks, and debris were increased hence increasing the adsorption of TBM.

This study clarified that the oxidation of microplastics during the water/wastewater treatment processes favored the adsorption of disinfection by-products, illustrating that the combination of these two emerging micropollutants caused greater risks to the ecological environment and human health. In future work, a study of raw water and the variety of MPs in material and size should be conducted.

## Figures and Tables

**Table 1:** The result of XPS of pristine PET and oxidized PETs.

Sample	Binding Energy (eV)	Half-Width(eV)	Peak Area	Area Percentage (%)	Ground
Pristine PET	531.5199	1.36	5048.592	27.26	-C=O
	532.1599	1.5	10,607.8	57.27	-OH, O-C-O
	533.5499	1.31	2864.492	15.47	C-O-C=O
ozonized PET	531.74	1.39	11,764.37	58.30	-C=O
	532.4499	1.27	4027.378	19.96	-OH, O-C-O
	533.4499	1.2	4387.843	21.74	C-O-C=O
ozonized-chlorinated PET	531.12998	1.35	3242.759	18.07	-C=O
	531.9599	1.27	10,664.25	59.44	-OH, O-C-O
	533.2099	1.37	4034.377	22.49	C-O-C=O
Pristine PET+TBM	531.57	1.28	5018.695	27.50	-C=O
	532.2899	1.68	10,229.62	56.06	-OH, O-C-O
	533.6699	1.2	2999.607	16.44	C-O-C=O
ozonized PET+TBM	531.6399	1.26	8662.442	42.61	-C=O
	532.6299	1.44	8070.743	39.70	-OH, O-C-O
	533.78999	1.16	3594.756	17.68	C-O-C=O
ozonized-chlorinated PET+TBM	531.61998	1.34	7419.59	43.62	-C=O
	532.399	1.52	6174.726	36.30	-OH, O-C-O
	533.58998	1.33	3416.825	20.09	C-O-C=O
ozonized-chlorinated PET+TBM	531.61998	1.34	7419.59	43.62	-C=O
	532.399	1.52	6174.726	36.30	-OH, O-C-O
	533.58998	1.33	3416.825	20.09	C-O-C=O

**Table 2:** Kinetic model parameters of pristine PET and oxidized PET.

Sample	Pseudo-First-Order Model			Pseudo-Second-Order Model		
	K_1_	Qe μg/μg	R^2^	K_2_	Qe μg/μg	R^2^
Pristine PET	0.285	2.486 × 10^−6^	0.9381	16111	2.667 × 10^−6^	0.9332
ozonized PET	0.128	2.842 × 10^−6^	0.9234	45552	2.959 × 10^−6^	0.8717
ozonized−chlorinated PET	0.0593	3.765 × 10^−6^	0.9343	116381	1.840 × 10^−6^	0.7779

**Table 3:** List of TBM sorption isotherm parameters.

Sample	Freundlich			Langmuir			D-R		
	k	1/n	R^2^	q_m_	b	R^2^	Q_max_	k	R^2^
Pristine PET	1.1 × 10^−7^	0.879	0.9789	1.720 × 10^−5^	0.0051	0.9835	1.707 × 10^−6^	7.939 × 10^−6^	0.8012
ozonized PET	1.712 × 10^−7^	0.754	0.7228	2.388 × 10^−6^	0.0736	0.5190	1.627 × 10^−6^	4.91 × 10^−6^	0.2330
ozonized-chlorinated PET	1.592 × 10^−10^	3.222	0.7684	−3.484 × 10^−8^	−0.0584	0.7599	3.703 × 10^−6^	2.81 × 10^−5^	0.9660

## Data Availability

Not available.
